# Binding of β_L_-Crystallin with Models of Animal and Human Eye Lens-Lipid Membrane

**DOI:** 10.3390/ijms241713600

**Published:** 2023-09-02

**Authors:** Preston Hazen, Geraline Trossi-Torres, Nawal K. Khadka, Raju Timsina, Laxman Mainali

**Affiliations:** 1 Biomolecular Sciences Graduate Programs, Boise State University, Boise, ID 83725, USA; prestonhazen@u.boisestate.edu (P.H.); geralinetrossito@u.boisestate.edu (G.T.-T.); 2 Department of Physics, Boise State University, Boise, ID 83725, USA; nawalkhadka@boisestate.edu (N.K.K.); timsinaraju47@gmail.com (R.T.)

**Keywords:** β_L_-crystallin, percentage of membrane surface occupied (MSO), maximum percentage of membrane surface occupied (MMSO), binding affinity (K_a_), mobility parameter, maximum splitting, hydrophobicity, cholesterol, cholesterol bilayer domains, EPR spin-labeling method, cataracts

## Abstract

Several discoveries show that with age and cataract formation, β-crystallin binds with the lens membrane or associates with other lens proteins, which bind with the fiber cell plasma membrane, accompanied by light scattering and cataract formation. However, how lipids (phospholipids and sphingolipids) and cholesterol (Chol) influence β-crystallin binding to the membrane is unclear. This research aims to elucidate the role of lipids and Chol in the binding of β-crystallin to the membrane and the membrane’s physical properties (mobility, order, and hydrophobicity) with β-crystallin binding. We used electron paramagnetic resonance (EPR) spin-labeling methods to investigate the binding of β_L_-crystallin with a model of porcine lens-lipid (MPLL), model of mouse lens-lipid (MMLL), and model of human lens-lipid (MHLL) membrane with and without Chol. Our results show that β_L_-crystallin binds with all of the investigated membranes in a saturation manner, and the maximum parentage of the membrane surface occupied (MMSO) by β_L_-crystallin and the binding affinity (K_a_) of β_L_-crystallin to the membranes followed trends: MMSO (MPLL) > MMSO (MMLL) > MMSO (MHLL) and K_a_ (MHLL) > K_a_ (MMLL) ≈ K_a_ (MPLL), respectively, in which the presence of Chol reduces the MMSO and K_a_ for all membranes. The mobility near the headgroup regions of the membranes decreases with an increase in the binding of β_L_-crystallin; however, the decrease is more pronounced in the MPLL and MMLL membranes than the MHLL membrane. In the MPLL and MMLL membranes, the membranes become slightly ordered near the headgroup with an increase in β_L_-crystallin binding compared to the MHLL membrane. The hydrophobicity near the headgroup region of the membrane increases with β_L_-crystallin binding; however, the increase is more pronounced in the MPLL and MMLL membranes than the MHLL membrane, indicating that β_L_-crystallin binding creates a hydrophobic barrier for the passage of polar molecules, which supports the barrier hypothesis in cataract formation. However, in the presence of Chol, there is no significant increase in hydrophobicity with β_L_-crystallin binding, suggesting that Chol prevents the formation of a hydrophobic barrier, possibly protecting against cataract formation.

## 1. Introduction

The eye lens is primarily composed of fiber cells that predominantly consist of structural crystallin proteins [1,2,3,4]. Crystallin proteins (α-, β-, and γ-crystallin) comprise approximately 90% of soluble lens proteins and mainly help develop and maintain the structure, transparency, and refractivity of the lens [5]. α-Crystallin primarily functions as a chaperone protein, helping to prevent aggregation of misfolded or denatured proteins and maintain long-term lens transparency [2,4,6]. On the other hand, β- and γ-crystallins play a critical role in maintaining the lens’s structure and refractivity [7]. These functions of crystallins are crucial for preventing cataract development [8]. However, with age and cataract formation, α- and β-crystallins associate with the lens membrane, decreasing the crystallin content in the cytoplasm and affecting membrane integrity and cataract formation [1,9,10,11,12,13]. The binding of crystallin proteins to the lens membrane in aged lenses is believed to be the mechanism of crystallins’ insolubilization [13,14,15]. It has been reported that β-crystallin is associated with other lens proteins, forming a higher molecular weight (HMW) protein [16,17,18,19,20], which further binds with the lens membrane [21], resulting in light scattering and cataract development. The amount of β-crystallin in HMW proteins is more prominent in cataractous lenses than in age-matched transparent lenses [19]. It has been reported that β-crystallin binds to membranes isolated from the human lens’s cortical and nuclear regions, and membrane-bound β-crystallin increases with age [1]. It is unclear how β-crystallin or HMW protein bind to the lens membrane, leading to cataract formation.

The binding of α-crystallin to the membrane has been investigated by our laboratory [12,22,23,24,25,26,27,28] and several others [18,21,29,30,31,32,33,34] to understand the mechanistic interaction at the molecular level; however, knowledge of the molecular-level mechanistic interaction involved in β-crystallin’s binding to the lens membrane is limited [9]. Previously, Zhu et al. [9] investigated the interaction of β-crystallin with membranes made of dihydrosphingomyelin (DHSM) using the fluorescence method and found that the order of the membrane’s headgroup is affected by β-crystallin binding. It has been reported, using the Laurdan and confocal microscopy approach, that the head group environment of the nuclear lens membrane changes with age, and β-crystallin might play a role in modulating this effect [9]. The lens lipid (phospholipids (PLs) and sphingolipids) and cholesterol (Chol) composition in the lens membrane changes significantly with age and cataracts [35,36,37,38,39,40], among species [35,36,41,42], and the location of the lens [39]. Sphingomyelin (SM), DHSM, phosphatidylcholine (PC), phosphatidylserine (PS), and phosphatidylethanolamine (PE) are the major lipids that build the fiber cells’ plasma membrane of the eye lens [37,41]. With increasing human age, the PC content declines with a corresponding increase in sphingolipids (SM and DHSM) content in the membrane [32,43]. It has also been reported that the PC lipid content is dominant in the eye lens membranes of lower lifespan animals (like porcine and mice); in contrast, sphingolipids are dominant in the human eye lens membrane [41]. The fiber cell plasma membrane of the eye lens has an exceptionally high level of Chol content, with a Chol/lipid molar ratio as high as ~4 [39,44]; however, the typical Chol/lipid molar ratio in the plasma membrane of other tissue and organs is between 0.1 and 0.5 [45,46]. Also, the Chol content in the lens membrane increases with age and is greater in nuclear membranes than cortical membranes [39,47], and the amount of Chol in the cataractous lens membrane is significantly lower than that found in age-matched transparent lenses [46,47]. Previous studies reported that the Chol/lipid molar ratio in porcine lens cortical and nuclear membranes are 0.6 and ~2 to 2.7, respectively [48,49], and the Chol/lipid ratio for mouse lens membranes is ~1 [41,48]. For the transparent human lens membrane of donor age groups 0–20, 21–40, 41–60, and 61–70 years, the Chol/lipid molar ratios are 0.6, 1.0, 1.4, and 1.8 in cortical membranes and 0.7, 1.2, 2.1, and 4.4 in nuclear membranes, respectively [39].

How the lipid and Chol composition influences β-crystallin binding to the membrane and how such binding may modulate the membrane’s physical properties (mobility, order, and hydrophobicity) is unclear, which we investigated in this manuscript. Also, β-crystallin exists as a dimer and higher-level oligomer [50], and the subunits between β-crystallin oligomers interact with each other via hydrophobic interactions [51]; however, the nature of the interaction between β-crystallin and the membrane remains unclear, which we have elucidated in this research. It has also been reported that with increasing human age, there is the development of a barrier for the transport of water molecules and antioxidants (glutathione) in the lens, which, over time, might promote protein oxidation, leading to cataracts [16,52]. It has been suggested that the binding of crystallins in an aged human lens membrane [15] occludes membrane pores and creates a barrier to the diffusion of polar molecules, contributing to nuclear cataract formation [14,53,54]. Whether the binding of β-crystallin to the membrane creates the hydrophobic barrier near the membrane surface and whether Chol modulates such a hydrophobic barrier are unclear, which we investigated in this study.

Electron paramagnetic resonance (EPR) is a powerful tool that can simultaneously provide information on the binding of β-crystallin with the membrane and the changes in the physical properties of the membranes with β-crystallin binding. In this approach, the cholesterol analog, cholestane spin-label (CSL), is incorporated into the membrane, and this spin-label monitors the binding of β-crystallin to the membrane. Our lab developed EPR approaches to obtain the percentage of membrane surface occupied (MSO) and maximum percentage of membrane surface occupied (MMSO) by α-crystallin, the binding affinity (K_a_) of α-crystallin to membranes, and the membranes’ physical properties (mobility, order, and hydrophobicity) with α-crystallin binding [12,22,23,24,25,27,28]. In this study, we used these developed EPR methods to investigate the binding of β_L_-crystallin (low molecular weight β-crystallin) with a model of human lens-lipid (MHLL), model of porcine lens-lipid (MPLL), and model of mouse lens-lipid (MMLL) membranes with 23 mol% Chol and without Chol. We estimated the MSO and MMSO by β_L_-crystallin and K_a_ of β_L_-crystallin to these membranes. We also estimated the physical properties (i.e., mobility, order, and hydrophobicity) near the headgroup regions of the membranes with β_L_-crystallin binding. The results of this study identify the role of the lipid composition and Chol composition in β_L_-crystallin binding to the membranes, the nature of the interaction between β_L_-crystallin and the membranes, and the modulation in the mobility, order, and hydrophobicity near the headgroup regions of the membrane with β_L_-crystallin binding.

## 2. Results

### 2.1. MSO by β_L_-Crystallin in Models of Human, Porcine, and Mouse Lens-Lipid Membrane

**Figure 1:**
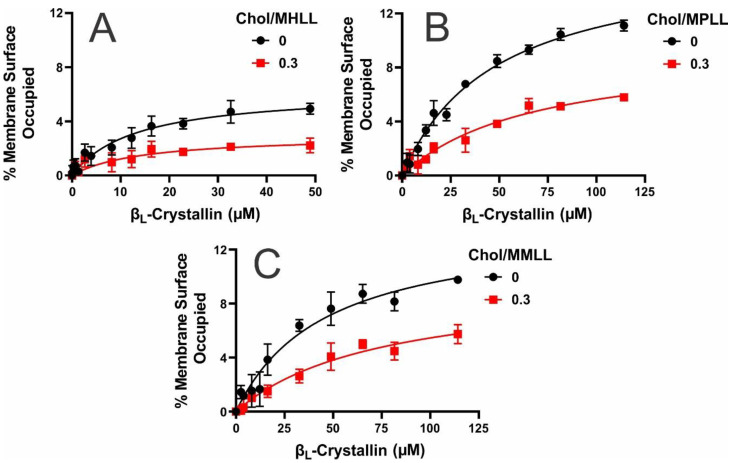
The percentage of membrane surface occupied (MSO) by β_L_-crystallin plotted as a function of the β_L_-crystallin concentration at Chol/lipid mixing ratios of 0 (black lines) and 0.3 (red lines). (**A**) The plot of the MSO by β_L_-crystallin for the Chol/MHLL membrane shows a maximum percentage of the membrane surface occupied (MMSO) of approximately 4.9% without Chol and 2.2% with Chol. (**B**) The plot of the MSO by β_L_-crystallin for the Chol/MPLL membrane shows an MMSO of approximately 11.1% without Chol and 5.8% in the presence of Chol. (**C**) The plot of the MSO by β_L_-crystallin for the Chol/MMLL membrane showed an MMSO of approximately 9.8% without Chol and 5.7% with Chol. The human, porcine, and mouse model membranes were incubated with varying concentrations of β_L_-crystallin (MHLL: 0–52.6 μM; MPLL and MMLL: 0–114.2 μM) for 16 h at 37 °C, and the EPR measurements were recorded at 37 °C. All results are the mean ± standard deviation (σ) of at least three independent experiments.

Depicted in Figure 1A–C is the MSO by β_L_-crystallin, shown as a function of the β_L_-crystallin concentration, for the Chol/MHLL, Chol/MPLL, and Chol/MMLL membranes at mixing ratios of 0 (black lines, without Chol) and 0.3 (red lines, with 23 mol% Chol). The MSO varied among the models, but all showed a positive, nonlinear relationship with the β_L_-crystallin concentration. Therefore, with an increased β_L_-crystallin concentration, each model showed an increase in the MSO before saturating at each MMSO. These data indicate that β_L_-crystallin associated in a saturable manner with each model’s membrane. In the absence of Chol, the MMSO among the three models significantly varied, with statistical significance set at *p* ≤ 0.05, at 4.9%, 11.1%, and 9.8% for the MHLL, MPLL, and MMLL membranes, respectively. Therefore, the MMSO, in the absence of Chol, followed the trend MMSO (MPLL) > MMSO (MMLL) > MMSO (MHLL). This variance in the MMSO by β_L_-crystallin is most likely explained by the differences in the lipid compositions among the models. The MHLL membrane consists of 66% SM, 11% 1-palmitoyl-2-oleoyl-sn-glycero-3-phosphatidylcholine (POPC), 8% 1-palmitoyl-2-oleoyl-sn-glycero-3-phosphatidylserine (POPS), and 15% 1-palmitoyl-2-oleoyl-sn-glycero-3-phosphoethanolamine (POPE). Similarly, the MPLL membrane consists of 29% SM, 35% POPC, 21% POPS, and 12% POPE, and the MMLL membrane consists of 15% SM, 46% POPC, 17% POPS, and 17% POPE. The lipid compositions of the MHLL, MPLL, and MMLL membranes were taken from previous studies [24,41]. While each membrane differs in the composition of SM, POPC, POPS, and POPE, the primary points of variation in the compositions and what was found to be most the impactful on β_L_-crystallin binding are the SM and POPC compositions. Specifically, β_L_-crystallin association with the membrane seems to primarily increase with an increase in the POPC content and, conversely, decrease with an increase in the SM content, in turn, explaining the increased MMSO seen in the MPLL and MMLL models, as they had the highest levels of POPC, and the lowest MMSO in the MHLL membrane, as it had the highest levels of SM relative to the two animal models. Therefore, the lipid composition strongly modulates β_L_-crystallin binding, resulting in the significant MMSO variation among all three models.

Interestingly, while these data align with our previous studies showing that lipid composition strongly affects α-crystallin membrane binding [24,27,28], β_L_-crystallin membrane binding strongly differs from α-crystallin membrane binding. In α-crystallin, we previously found increased binding to SM and in the SM-predominant MHLL membrane with lower levels of binding to PLs (POPC, POPS, and POPE) and the PL-dominant MPLL and MMLL membranes [24,27], while we see the opposite effect in β_L_-crystallin binding to membranes. Moreover, in our prior studies on α-crystallin binding to the model lens-lipid membranes [24], the MMSO by α-crystallin (i.e., binding saturation) in all of the models (MHLL, MPLL, and MMLL) was achieved with α-crystallin concentrations ≤ 52.6 μM. However, in the case of β_L_-crystallin, saturation was achieved in the MHLL membrane by 52.6 μM β_L_-crystallin (Figure 1A), but saturation was not achieved in the MPLL or MMLL membranes with 52.6 μM and did appear to saturate at ~114.2 μM (Figure 1B,C). These observations suggest that the lipid composition determines the concentration of β_L_-crystallin needed to achieve the MMSO, i.e., binding saturation. Also, the difference in α- and β_L_-crystallin concentrations required to achieve binding saturation in the MPLL and MMLL membranes suggests that the lipid composition modulates the α-crystallin and β_L_-crystallin binding to the membranes differently. Moreover, β_L_-crystallin consists of primary dimers and trimers (average molecular weight of a single subunit: ~24 kDa) [55], whereas α-crystallin exists as larger oligomers of ~40 subunits (average molecular weight of a single subunit: ~20 kDa) [12]; these difference in the oligomeric size and the amino acid residues interacting with the membrane might result in different α- and β_L_-crystallin concentrations needed to achieve the MMSO.

However, while SM and POPC are the dominant factors affecting β_L_-crystallin binding with the membrane, this trend is not exact, as the highest MMSO was seen in the MPLL membrane, which had the 2nd highest levels of POPC and SM. In contrast, the MMLL membrane contained the highest amount of POPC and the lowest amount of SM, yet only showed the 2nd highest MMSO. Therefore, if the trend was exact, the MMLL membrane should show the highest MMSO. So, while they do not appear to be as impactful, the POPS and POPE levels in each membrane may be the reason for the deviation from the trend. The POPS levels follow a MPLL > MMLL > MHLL trend, while the POPE levels in each model are MMLL > MHLL > MPLL. Therefore, the MPLL membrane had the highest levels of POPS, while the MHLL membrane showed the lowest, which is the same trend seen for the MMSO. Moreover, the MMLL and MHLL membranes showed higher POPE levels and a lower MMSO than the MPLL membrane. Based on this information, it appears that increased levels of POPS appear to correlate with a possible increase in β_L_-crystallin binding, and, conversely, increased levels of POPE may diminish binding to the membrane. Interestingly, these results align with our previous study on α-crystallin interactions with individual lipids, where POPS showed intermediate levels of binding, but POPE was shown to diminish α-crystallin binding significantly [24,28]. In turn, while the effects of POPS are unclear, POPE may inhibit both α- and β_L_-crystallin association with eye lens-lipids membranes. Therefore, individual lipids have shown a strong influence on β_L_-crystallin interactions with the membrane, but the synergistic effect of POPS and POPE with POPC and SM is likely the reason that the MMSO seen in each model does not directly correlate with the SM and POPC levels.

In addition to the lipid composition impacting on the MSO by β_L_-crystallin, adding Chol further affected the binding of β_L_-crystallin to all three models’ lens-lipid membranes. As shown by the red line in Figure 1A–C, adding Chol significantly decreased the MSO of β_L_-crystallin in each of the three models (*p* ≤ 0.05). Specifically, the MMSO for the Chol/MHLL, Chol/MPLL, and Chol/MMLL membranes decreased to 2.2%, 5.8%, and 5.7%, respectively. Therefore, regardless of the lipid composition, Chol integrating into the membrane consistently inhibits the binding of β_L_-crystallin to the membrane. Our previous studies on α-crystallin membrane binding show that Chol inhibits the binding of α-crystallin to the membrane [22,24,25]. Therefore, Chol appears to inhibit the binding of both α-crystallin and β_L_-crystallin to the membrane, and this inhibitory role of Chol in the lens membrane might be crucial for maintaining the lens membrane and cytoplasm homeostasis by inhibiting β_L_-crystallin binding and increasing the concentration of β_L_-crystallin in the cytoplasm of the lens to preserve the structure and refractive properties of the lens and, likely, to protect against the development and progression of cataracts.

### 2.2. K_a_ of β_L_-Crystallin to the Models’ Lens-Lipid Membranes and the Effects of Chol

**Figure 2:**
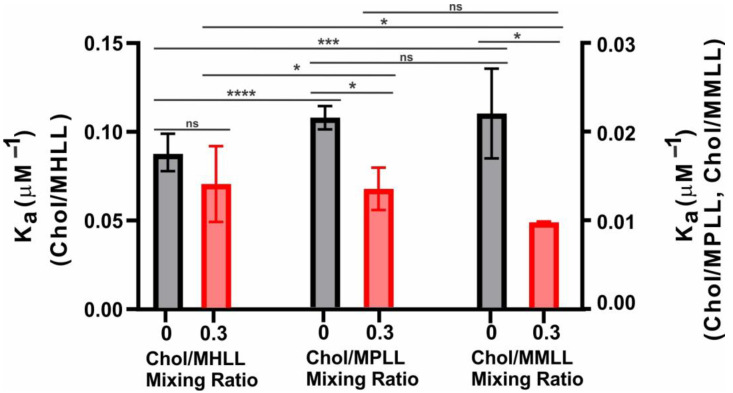
Binding affinity (K_a_) of β_L_-crystallin to the Chol/MHLL (left), Chol/MPLL (middle), and Chol/MMLL (right) membranes at Chol/lipid mixing ratios of 0 (black) and 0.3 (red). As depicted on the left y-axis, the Chol/MHLL membrane had a K_a_ of 0.08 ± 0.01 μM^−1^ without Chol (black) and 0.07 ± 0.02 μM^−1^ with Chol (red). Shown along the right y-axis, the K_a_ of β_L_-crystallin to the Chol/MPLL membrane was 0.02 ± 0.001 μM^−1^ without Chol (black) and 0.014 ± 0.002 μM^−1^ with Chol (red). Further, using the right y-axis, the binding affinity of β_L_-crystallin is shown for the Chol/MMLL membrane with an affinity of 0.02 ± 0.005 μM^−1^ without Chol (black) and 0.01 ± 0.0001 μM^−1^ with Chol (red). The K_a_ in all models decreased with the addition of Chol, indicating that the addition of Chol within the membrane decreases the affinity of β_L_-crystallin binding to the membranes. The results are the mean ± standard deviation (σ) from at least three independent experiments. *, *** and **** represent a *p*-value < 0.05, <0.001, and <0.0001, respectively. “ns” represents not significant.

Depicted in Figure 2 is the K_a_ of β_L_-crystallin to the Chol/MHLL, Chol/MPLL, and Chol/MMLL membranes at mixing ratios of 0 and 0.3. The K_a_ of β_L_-crystallin for each model membrane without Chol followed the trend K_a_ (MHLL) > K_a_ (MMLL) ≈ K_a_ (MPLL), with the MHLL membrane’s K_a_ being significantly higher than that of the MPLL (*p* < 0.0001) and MMLL (*p* = 0.0003) membranes, and there was no significant difference between the K_a_ of the MPLL and MMLL membranes (*p* = 0.2804). This variance in the K_a_ is likely due to the variation in the lipid compositions among the models, altering the capacity of the membrane to modulate interactions with β_L_-crystallin. Interestingly, without Chol, the MHLL membrane showed the highest K_a_, and the MPLL membrane showed the lowest; yet, as previously discussed, the MHLL membrane showed the lowest MMSO, while the MPLL membrane had the highest MMSO by β_L_-crystallin. This contrast between K_a_ and MMSO is likely because the lower MSO and MMSO in the MHLL membrane may allow for a faster binding saturation. In contrast, the higher MMSO in the MPLL membrane is reached at a slower rate and saturates at a higher β_L_-crystallin concentration, resulting in a higher K_a_ in the MHLL membrane relative to the two animal models. Moreover, the difference between the K_a_ and MMSO may also be because β_L_-crystallin has a higher affinity for SM relative to POPC, but the structure and resulting membrane structure of SM-dominant membranes may prevent further binding of β_L_-crystallin, while the membrane structure of POPC-dominant membranes allows for increased binding. Moreover, there is likely a synergistic effect among lipids that alters the affinity of β_L_-crystallin to the distinct lipid composition of each model. Regardless, these data indicate that β_L_-crystallin does differentially bind and has a varying affinity for the lipid composition in each eye lens-lipid model membrane.

As additionally seen in Figure 2, adding Chol altered the K_a_ in all three analyzed models, making the trend MHLL > MPLL ≈ MMLL. Similarly, the K_a_ in the MHLL membrane remained significantly higher than in the MPLL (*p* = 0.0147) and MMLL (*p* = 0.0118) membranes, but there was no significant difference (*p* = 0.1930) in the K_a_ between the MPLL and MMLL membranes. Moreover, adding Chol explicitly reduced the K_a_ in the three models, with the change being the largest in the MPLL and MMLL membranes. The decrease in the K_a_ with the addition of Chol was statistically significant in the MPLL (*p* = 0.0229) and MMLL (*p* = 0.0472) membranes, whereas the decrease in the K_a_ with the addition of Chol in the MHLL membrane was not statistically significant (*p* = 0.1970). This decrease in the K_a_ shows that the addition of Chol lowers the affinity of β_L_-crystallin to the membrane, which follows a similar trend as our prior studies on α-crystallin membrane interactions, where the addition of Chol significantly reduced the affinity and binding of α-crystallin to a membrane [24,25]. Conversely, this reduction in affinity seen with the addition of Chol may, therefore, also be a reason for the decrease in the MSO seen with the addition of Chol, as shown in Figure 1A–C, and the more significant reduction in the MSO seen in the MPLL and MMLL membranes may directly correlate with their larger reduction in the K_a_ relative to those seen in the MHLL membrane. Ultimately, these data show that adding Chol to a model eye lens-lipid membrane reduces the affinity for binding of β_L_-crystallin near the headgroup region of the membrane.

### 2.3. Mobility near the Surface of the Model Lens-Lipid Membranes with Chol and β_L_-Crystallin Binding

**Figure 3:**
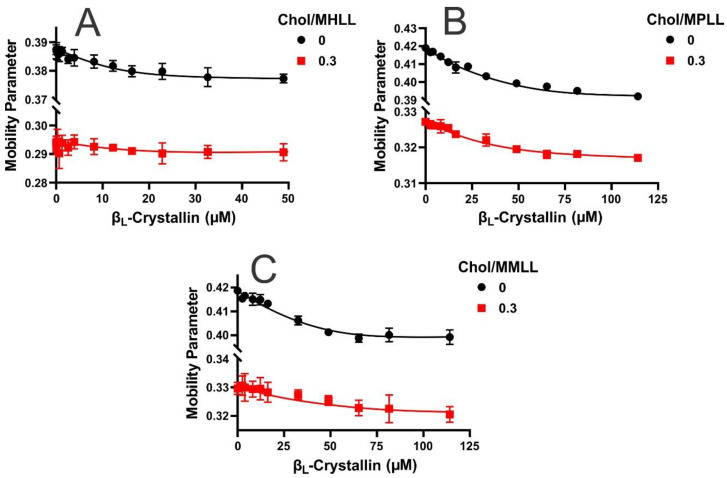
(**A**–**C**) The mobility parameter for the Chol/MHLL, Chol/MPLL, and Chol/MMLL membranes at mixing ratios of 0 (black line) and 0.3 (red line), respectively, obtained at 37 °C and plotted as a function of the β_L_-crystallin concentration. In the absence of Chol, the mobility parameter decreased with an increase in the β_L_-crystallin concentration for all of the membrane systems investigated. The presence of Chol decreased the mobility parameters for all of the membrane systems investigated. For the Chol-containing membranes (red line in (**A**–**C**)), the mobility parameter decreased with an increase in the β_L_-crystallin concentration for the Chol/MPLL and Chol/MMLL membrane; however, the decrease was not significant for the Chol/MHLL membrane. The decrease in the mobility parameter with β_L_-crystallin binding indicates that the membrane becomes immobilized near the headgroup regions. The results are the mean ± standard deviation (σ) of at least three independent experiments.

The mobility parameter gives the orientational and rotational dynamics of the cholesterol analog spin-label (CSL) in the membrane [56,57,58], which also provides information concerning mobility near the headgroup regions with protein binding [27,28]. Shown in Figure 3A–C are the mobility parameter profiles for the Chol/MHLL, Chol/MPLL, and Chol/MMLL membranes displayed as a function of the β_L_-crystallin concentration. Among all three models, the highest mobility parameter values were found for the membranes without Chol and β_L_-crystallin. Moreover, the mobility parameter for the MPLL and MMLL membranes was higher than that of the MHLL membrane due to the variation in the lipid composition of each model, with the MHLL membrane being composed predominantly of SM, and SM has been shown to reduce membrane mobility [24,25]. The MPLL and MMLL membranes are composed primarily of POPC and have similar but significantly higher mobility parameter values than the MHLL membrane. Therefore, it appears that the lipid composition of each membrane alters the relative mobility in the absence of β_L_-crystallin and Chol, with an increase in the POPC content correlating to the increase in mobility, and an increase in SM leading to a reduction in the membrane’s mobility near the headgroup region.

As seen by the black lines in Figure 3A–C, the addition of β_L_-crystallin in all three lens-lipid membrane models in the absence of Chol resulted in a reduction in mobility, indicating that the binding of β_L_-crystallin reduces the mobility of the membrane near the headgroup region. The overall decrease in the mobility parameter for the MHLL, MPLL, and MMLL membranes with β_L_-crystallin binding was statistically significant at *p* ≤ 0.05. This information, together with the MSO data previously discussed in Figure 1A–C, indicates that the larger binding (i.e., larger MMSO) of β_L_-crystallin near the surface of the membrane is correlated with the corresponding larger decrease in mobility near the headgroup regions of the membrane. Consequentially, the more considerable reductions in the mobility parameter seen with the addition of β_L_-crystallin to the MPLL membrane compared to the MMLL membrane were because of the MPLL membrane having a larger MSO than the MMLL membrane. Similarly, the MHLL membrane showed the smallest decrease in the mobility parameter relative to the two animal models and, conversely, had the lowest MSO. Furthermore, the addition of Chol also reduced the mobility parameter values seen in each model relative to each model’s Chol-free samples, and the decrease was statistically significant (*p* ≤ 0.05). This reduction in the mobility parameter indicates that the area near the membrane headgroup region becomes immobilized with the addition of Chol.

While the mobility of each sample was reduced with the addition of Chol, the relative trend remained the same with the addition of β_L_-crystallin in each of the three model lens-lipid membranes, leading to an apparent reduction in the mobility parameters. However, in the presence of Chol, the overall decrease in the mobility parameter was larger for the Chol/MPLL and Chol/MMLL membranes compared to the Chol/MHLL membrane. The overall decrease was statistically significant at *p* ≤ 0.05 for the Chol/MPLL and Chol/MMLL membranes, but the difference was no longer statistically significant in the Chol/MHLL membrane. While the addition of β_L_-crystallin still caused a decrease in the mobility parameter of the Chol-containing samples, the reduction from the control to the highest β_L_-crystallin concentration was smaller than the decrease seen in the Chol-free counterparts for each model. Therefore, the reduction in β_L_-crystallin binding due to the addition of Chol, as seen in Figure 1A–C, consequentially reduced the impact β_L_-crystallin had on the mobility parameter for each model’s membrane. This further verifies that the association of β_L_-crystallin with the membrane results in a reduction in the membrane mobility, and the addition of Chol reduces the binding of β_L_-crystallin, ultimately decreasing the effects of β_L_-crystallin on mobility near the headgroup regions of the membrane.

### 2.4. Order Below the Surface of Model Lens-Lipid Membranes with the Addition of Chol and β_L_-Crystallin Binding

**Figure 4:**
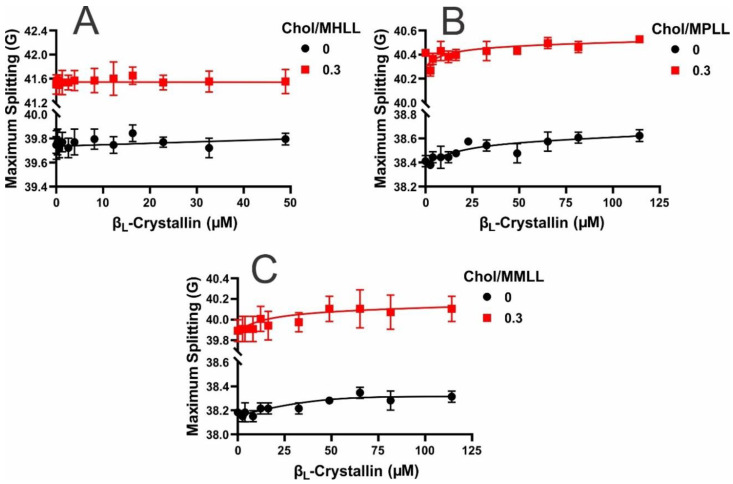
The maximum splitting profiles obtained at 37 °C using the CSL spin-labels within the models of human, porcine, and mouse lens-lipid membranes plotted as a function of the β_L_-crystallin concentration. (**A**) The maximum splitting measurements of the MHLL membranes (black line) showed possible slight increases with an increasing β_L_-crystallin concentration that were not statistically significant. With the addition of Chol at a Chol/MHLL mixing ratio of 0.3, there was no significant change in the maximum splitting with the addition of β_L_-crystallin. (**B**,**C**) Both in the presence and absence of Chol, the maximum splitting measurements of the MPLL (**B**) and MMLL (**C**) membranes showed slight increases with an increasing β_L_-crystallin concentration. The slight increases seen with the binding of β_L_-crystallin imply that, while it does not have a substantial effect, the binding of β_L_-crystallin causes a slight increase in the order of the membrane near the headgroup region. Moreover, the increase in the maximum splitting seen in the Chol-containing MHLL, MPLL, and MMLL membranes relative to those without Chol indicates the headgroup region of the membrane becomes more ordered with the addition of Chol. The results are the mean ± standard deviation (σ) of at least three independent experiments.

The maximum splitting is a parameter related to the order parameter that provides the amplitude of the wobbling motion of the long axis of the CSL spin-label in the membrane [57,58,59], which also provides information concerning the order near the headgroup region with protein binding [25,28]. Figure 4A–C show the maximum splitting profiles as a function of the β_L_-crystallin concentration for the Chol/MHLL, Chol/MPLL, and Chol/MMLL membranes, respectively. In the absence of Chol and β_L_-crystallin, the maximum splitting values for each model membrane followed the trend: MHLL > MPLL > MMLL. Meaning without Chol, the MHLL membrane had the highest level of order, while the MMLL membrane was the least ordered near the membrane’s headgroup region, indicating that the lipid composition modulates the membrane order, with increased levels of SM correlating with an increase in the order relative to the PL-predominant membranes, as described previously [24,25]. Interestingly, without Chol, the addition of β_L_-crystallin to each model lens-lipid membrane appears to have caused slight increases in the maximum splitting values. This increase in the order was most noticeable in the MPLL and MMLL membranes and very slight in the MHLL membrane; however, the overall increase in order was statistically significant, with a *p* ≤ 0.05 only for the MMLL membranes. These data, therefore, indicate that the binding of β_L_-crystallin may cause a slight increase in the order but does not, generally, have a significant impact on the order of the membrane near the surface. Moreover, as the MMSO was the highest in the two lowest ordered membranes, MPLL and MMLL, the reduced ordered membranes appear to facilitate more association of β_L_-crystallin near the headgroup region than the highly ordered SM-dominant MHLL membrane. Interestingly, our previous research shows that the association of α-crystallin was greater in ordered MHLL membranes [24]. Also, during aging, the content of the order lipids (sphingolipids) increases with age [32,43]. These observations suggest that with aging, the association of α-crystallin with the lens membrane might be more favorable than that of β_L_-crystallin [24].

This general trend in which the order of each membrane was MHLL > MPLL > MMLL and the addition of β_L_-crystallin caused slight increases in the maximum splitting values remained the same with the addition of Chol. However, the incorporation of Chol in each membrane significantly increased (*p* ≤ 0.05) the maximum splitting (i.e., order) values for each model. These data, again, aligns with the MMSO data and further verify that β_L_-crystallin decreased association with an increase in ordered membranes, as Chol increased the membrane order and, consequentially, reduced the MSO by β_L_-crystallin in all three models of lens-lipid membranes. Moreover, in the Chol-containing membranes, the binding of β_L_-crystallin was shown to cause slight increases in order amongst each model lens-lipid membrane; however, the MPLL membrane was the only membrane system that showed an overall increase in the order that was statistically significant (*p* ≤ 0.05). These data show that the integration of Chol into a model of the lens-lipid membrane causes significant increases in the order of the membrane near the headgroup region, whereas the binding of β_L_-crystallin causes slight increases in the order near the headgroup region of the membrane.

### 2.5. Hydrophobicity near the Surface of Model Lens-Lipid Membranes with the Addition of β_L_-Crystallin and Chol

**Figure 5:**
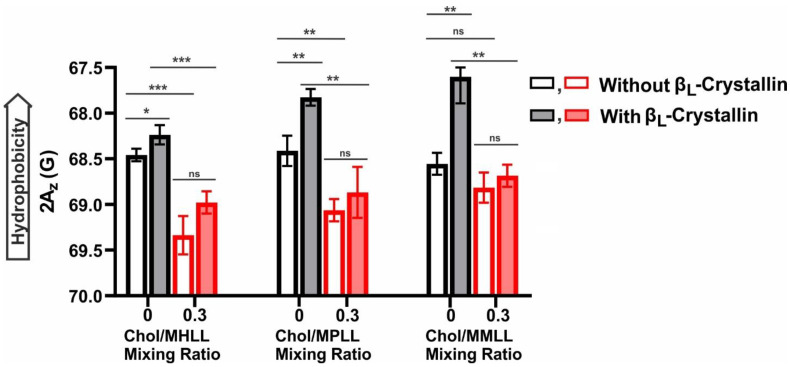
Hydrophobicity (2Az) near the headgroup region of each membrane was probed with the CSL spin-label within these membranes and plotted as a function of the β_L_-crystallin and Chol concentrations. Shown leftmost is the hydrophobicity data for the MHLL membrane with 52.6 μM β_L_-crystallin (shaded bars) and without β_L_-crystallin (clear bars) at Chol/MHLL mixing ratios of 0 (black) and 0.3 (red). At both mixing ratios, the hydrophobicity increased with the addition of β_L_-crystallin; however, the increase was only statistically significant (*p* = 0.0120) without Chol and was no longer statistically significant (*p* = 0.0531) in the presence of Chol. Shown in the middle and farthest right is the hydrophobicity data for the MPLL and MMLL membranes, respectively, with 114.2 μM β_L_-crystallin (shaded bars) and without β_L_-crystallin (clear bars) at Chol/MPLL and Chol/MMLL mixing ratios of 0 (black) and 0.3 (red). Both the Chol/MPLL and Chol/MMLL membranes showed a similar trend to the Chol/MHLL membrane, with the addition of β_L_-crystallin causing an increase in the hydrophobicity. Moreover, this increase in the hydrophobicity was, again, the largest in the absence of Chol, being statistically significant in both the Chol/MPLL (*p* = 0.0060) and Chol/MMLL membranes (*p* = 0.0046), making the increase more pronounced for the MPLL and MMLL membranes than the MHLL membranes. In the presence of Chol, the increase in the hydrophobicity with the addition of β_L_-crystallin was, again, no longer statistically significant for both the Chol/MPLL (*p* = 0.2085) and Chol/MMLL (*p* = 0.2109) membranes. In all three model lens-lipid membranes, with the addition of Chol, there was a reduction in the hydrophobicity, implying that Chol reduces the hydrophobic environment near the headgroup region of the membrane. The binding of β_L_-crystallin forms a hydrophobic barrier near the membrane surface; however, Chol prevents the formation of such a hydrophobic barrier. The results are the mean ± standard deviation (σ) of at least three independent experiments. *, ** and *** represent a *p*-value <0.05, <0.01, and <0.001, respectively. “ns” represents not significant.

Displayed in Figure 5 is the hydrophobicity near the headgroup regions of the Chol/MHLL, Chol/MPLL, and Chol/MMLL membranes at mixing ratios of 0 and 0.3. In each model lens-lipid membrane, the addition of β_L_-crystallin at its highest concentration (i.e., 52.6 μM β_L_-crystallin for the MHLL and 114.2 μM β_L_-crystallin for the MPLL and MMLL membranes) caused an apparent decrease in the 2A_z_ value, which corresponded to an increase in thee hydrophobicity. The highest hydrophobicity values for each model lens-lipid membrane were with β_L_-crystallin in the absence of Chol, with the MMLL membrane having the most hydrophobic environment near the surface of the membrane, followed by the MPLL and MHLL membranes. Therefore, the addition of β_L_-crystallin at its highest concentration resulted in a statistically significant increase in the hydrophobicity in the Chol-free MHLL (*p* = 0.0120), MPLL (*p* = 0.0060), and MMLL (*p* = 0.0046) membranes. However, the increase in the hydrophobicity was more pronounced in the MPLL and MMLL membranes than in the MHLL membrane. This pronounced increase in the hydrophobicity in the MPLL and MMLL membranes compared to the MHLL membrane with β_L_-crystallin binding can be explained on the basis of the differences in the order of the models of the lens-lipid membranes and the MMSO by β_L_-crystallin in the models of lens-lipid membranes. As discussed in Section 2.4, the binding of β_L_-crystallin was higher for the less-ordered MPLL and MMLL membranes than the ordered MHLL membranes. Also, as mentioned in Section 2.1, the MMSO by β_L_-crystallin was higher for the less-ordered MPLL and MMLL membranes than the ordered MHLL membrane, suggesting that a greater amount of β_L_-crystallin (i.e., more hydrophobic residues of β_L_-crystallin) are near the headgroup regions of the MPLL and MMLL membranes than the MHLL membrane, and, possibly, a larger number of water molecules were expelled around the headgroup regions of the MPLL and MMLL membranes than the MHLL membrane, resulting in the pronounced increase in hydrophobicity seen in the MPLL and MMLL membranes than the MHLL membrane (see last paragraph of Section 2.5, which explains the hydrophobic interactions of β_L_-crystallin with the models of lens-lipid membranes). This increase in the hydrophobicity indicates that β_L_-crystallin binding creates a hydrophobic barrier for the passage of polar molecules, which supports the barrier hypothesis in cataract formation.

Furthermore, in the presence of Chol, there was a statistically significant decrease in hydrophobicity relative to either the β_L_-crystallin-absent (Chol/MHLL: *p* = 0.0006, Chol/MPLL: *p* = 0.0060) or β_L_-crystallin-containing samples (Chol/MHLL: *p* = 0.0004, Chol/MPLL: *p* = 0.0038, and Chol/MMLL: *p* = 0.0041) for all three models of lens-lipid membranes, except for the β_L_-crystallin-absent Chol/ MMLL (*p* = 0.2940) membrane. This decrease in the hydrophobicity near the headgroup regions of the membranes with the addition of Chol was due to the separation of the polar headgroup regions of the membrane that resulted in increased water penetration near the headgroup regions with a corresponding increase in polarity (i.e., decrease in hydrophobicity) [24,60]. However, while all three models of lens-lipid membranes showed a decrease in hydrophobicity in the presence of Chol, the relative trend remained the same, with the β_L_-crystallin-containing samples for each model of lens-lipid membrane showing the highest levels of hydrophobicity relative to their β_L_-crystallin-free control counterpart (Figure 5). Moreover, while the relative trend remained with the addition of Chol, the change in the hydrophobicity seen between the controls (membranes without β_L_-crystallin) and the membranes containing the β_L_-crystallin samples was no longer statistically different for all three model lens-lipid membranes (Chol/ MHLL: *p* = 0.0531, Chol/MPLL: *p* = 0.2085, and Chol/MMLL: *p* = 0.2109). These results suggest that Chol prevents the formation of a hydrophobic barrier near the headgroup region of the membrane, possibly protecting against cataract formation.

This hydrophobicity data, paired with the information on the MSO (Figure 1A–C) and K_a_ (Figure 2), show that the association of β_L_-crystallin with the membrane is likely due to hydrophobic interactions. In each of the three β_L_-crystallin-containing and -absent model lens-lipid membranes, excluding the β_L_-crystallin-absent Chol/MMLL membrane, adding Chol resulted in a statistically significant (*p* ≤ 0.05) reduction in the hydrophobicity compared to the hydrophobicity levels seen in each model lens-lipid membrane without Chol. Relatedly, the addition of Chol, as previously discussed in Figure 1A–C, also resulted in a significant reduction in the MMSO by β_L_-crystallin across all three model lens-lipid membranes. Also, the addition of Chol reduced the K_a_ of β_L_-crystallin to all three model lens-lipid membranes, as previously described in Figure 2. Together, these data imply that Chol reduces the hydrophobicity near the surface of each model of the lens-lipid membrane, resulting in a corresponding inhibition of the binding of β_L_-crystallin with the membrane. These data indicate that the binding of β_L_-crystallin near the membrane surface is predominantly due to and regulated by hydrophobic interactions, and the reduction in the hydrophobicity seen with the addition of Chol, therefore, reduces the favorability of the binding of β_L_-crystallin to the models of lens-lipid membranes.

## 3. Discussion

Our results ultimately show that β_L_-crystallin appears to bind to eye lens-lipid membrane models, likely through hydrophobic interactions, and the unique lipid composition and Chol content of each model shows to strongly modulate the interactions of β_L_-crystallin with the individual models. The MHLL, MPLL, and MMLL membranes, consisting of SM, POPC, POPS, and POPE, significantly vary in their lipid compositions. The binding of β_L_-crystallin significantly varied among the three models due to the differences in the lipid compositions, with the SM levels inversely impacting on the β_L_-crystallin binding, while the POPC composition had a positive correlation with β_L_-crystallin binding. While these two components showed the most substantial impact on β_L_-crystallin binding, the POPE and POPS levels also had some effect on the interactions of β_L_-crystallin with the membranes, with increased POPS causing a possible increase in β_L_-crystallin binding, while inversely, increased levels of POPE may inhibit binding. We determined the K_a_ of β_L_-crystallin in each model and found it to follow the trend: MHLL > MMLL > MPLL; the exact inverse of the observed MMSO data. This contrast between the K_a_ and MMSO may occur because the lower MSO and MMSO in the MHLL membrane may allow for a faster binding saturation. In contrast, the higher MMSO in the MPLL and MMLL membranes is reached at a slower rate and saturates at a higher β_L_-crystallin concentration, resulting in a lower K_a_ in the MPLL and MMLL membranes relative to the MHLL membrane. These data are, therefore, indicative of the notion that β_L_-crystallin does bind differentially to models of human and animal eye lens-lipid membranes and varying eye lens-lipid compositions.

Moreover, the lipid composition and binding of β_L_-crystallin also showed to further impact the membrane’s physical properties. First, in the absence of β_L_-crystallin, the mobility of the membrane seems to be strongly affected by the lipid composition, with the POPC-dominant MPLL and MMLL membranes showing the highest mobility, while the SM-dominant MHLL membrane had significantly lower mobility near the headgroup region of the membrane. Moreover, the binding of β_L_-crystallin showed an impact and decrease on the mobility of a membrane directly, with a reduction in the mobility parameter being the inverse of the MMSO, with the highest levels of the MMSO resulting in the most significant decrease in the mobility parameter. Therefore, the addition and association of β_L_-crystallin with a membrane was shown to reduce the lipid mobility near the headgroup region of the membrane. In addition to membrane mobility, the order near the headgroup region of the membrane was also shown to be strongly affected by the lipid composition and slightly altered with β_L_-crystallin binding. Generally, although not significant, the addition of β_L_-crystallin to each model lens-lipid membrane caused possible slight increases in the order, with the most noticeable increases correlating with and being found in the models exhibiting the highest MMSO by β_L_-crystallin. Moreover, the data further verify the notion that β_L_-crystallin differentially binds to each of the three model membranes and, at varying levels, this binding was shown to inversely impact and decrease the mobility while slightly increasing the order of the membrane near the surface.

In addition to finding that β_L_-crystallin does bind to eye lens-lipid membrane models, which may further alter the physical properties of the membrane near the headgroup region, this study shows that the Chol content within a membrane has a significant impact on both the binding of β_L_-crystallin and the physical properties of the membrane. The addition of Chol, at a Chol/lipid mixing ratio of 0.3, significantly reduced the binding and MMSO by β_L_-crystallin in each of the three membranes (Chol/MHLL, Chol/MPLL, and Chol/MMLL). “Lipid rafts”, also called phase-separated microdomains, are formed in membranes typically rich in sphingolipids and Chol [61], and raft domains are believed to be in the liquid-ordered (*l*_o_) phase [62,63,64]. The phase diagram reported earlier for the Chol/SM and Chol/PC membrane system showed that the liquid-disordered (*l*_d_) phase plus *l*_o_ phase formed with a Chol content between ~8 mol% and ~28 mol% [65,66]. Based on these observations, our investigated Chol/MHLL, Chol/MPLL, and Chol/MMLL membranes with 23 mol% Chol might consist of *l*_o_ and *l*_d_ phases, and the Chol/MHLL membrane rich in SM content might contain raft domains in the *l*_o_ phase. The percentage decrease in the MMSO by β_L_-crystallin with the addition of Chol was larger in the case of the Chol/MHLL membrane compared to the Chol/MPLL and Chol/MMLL membranes, suggesting that the possible formation of raft domains in the SM-rich Chol/MHLL membrane decreases the MMSO more than the Chol/MPLL and Chol/MMLL membranes, where raft domains may not be formed. Moreover, adding Chol lowered the K_a_ of β_L_-crystallin in all three models, meaning the addition and integration of Chol into a model of lens-lipid membrane reduces the affinity and inhibits the binding of β_L_-crystallin to the membrane. This is significant, as the Chol content in a transparent human lens membrane increases with aging [39]. In contrast, the Chol content in a cataractous human lens membranes is significantly lower [44]. This reduction in Chol in a cataractous lens membranes likely promotes β_L_-crystallin binding, forming large crystallin–membrane aggregates that might be responsible for light scattering, ultimately aiding in the development and progression of cataracts. Adding Chol at a Chol/lipid mixing ratio of 0.3 caused both a significant increase in the order and a significant decrease in the mobility near the membrane surface of each of the three models relative to the Chol-free counterparts of each sample, and a similar effect of Chol was observed in our previous study [24]. Furthermore, in the presence of Chol, the addition of β_L_-crystallin showed a similar impact on the membrane’s physical properties to that seen without Chol, with β_L_-crystallin binding causing slight reductions in the membrane’s mobility and, possible, but insignificant increases in the membrane order. However, while the trend remained the same, the effects of β_L_-crystallin binding on the membrane’s surface mobility and order were reduced in the Chol-containing Chol/MHLL, Chol/MPLL, Chol/MMLL membranes relative to the effects of β_L_-crystallin binding seen in each of the model lens-lipid membranes without Chol. This change in impact is likely due to the reduction in β_L_-crystallin binding seen with the addition of Chol and further verifies both that β_L_-crystallin is binding to the membrane and that this binding is responsible for the changes seen, to varying degrees, in the mobility and order of each model lens-lipid membrane.

Lastly, these results reveal that the displayed interactions of β_L_-crystallin with the model of the lens-lipid membrane were likely due to the hydrophobic interactions modulated by the Chol and lipid composition. In the absence of Chol, the MHLL, MPLL, and MMLL membranes all showed a significant increase in the hydrophobicity with the addition of β_L_-crystallin. Moreover, the most notable increases in hydrophobicity were seen in the MPLL and MMLL membranes, where binding saturation was observed with a β_L_-crystallin concentration approximately three times larger in the MPLL and MMLL membranes than in the MHLL membrane. The MMSO by β_L_-crystallin in the MPLL and MMLL membranes was substantially higher than that of the MHLL membrane. Therefore, the more significant increase in hydrophobicity found in the MPLL and MMLL membranes was likely due to the increased MMSO by β_L_-crystallin and, conversely, the comparatively lower change in hydrophobicity seen in the MHLL membrane was likely due to the relatively lower MMSO by β_L_-crystallin. The higher MMSO by β_L_-crystallin likely expelled a greater number of water molecules around the headgroups regions, resulting in an increase in hydrophobicity (i.e., decrease in polarity) around the headgroup regions. The addition of Chol, both with and without β_L_-crystallin, also had a significant impact on the hydrophobic environment near the surface of each membrane, causing significant decreases in hydrophobicity in all three analyzed models, except for Chol/MMLL membranes without β_L_-crystallin, where the decrease was not statistically significant. Therefore, the addition of Chol in the Chol/MHLL, Chol/MPLL, and Chol/MMLL membranes likely separates the polar headgroups, which increases water penetration near the membrane surface [25] and, consequentially, reduces the hydrophobicity around the membrane headgroup region. The incorporation of Chol into the model lens-lipid membrane reduces the hydrophobicity near the membrane surface, resulting in the corresponding decrease in the MMSO by β_L_-crystallin and K_a_ of β_L_-crystallin to each model lens-lipid membrane. This shows that the β_L_-crystallin binding to the membrane was likely due to hydrophobic interactions near the headgroup region of the membrane. Ultimately, this indicates that Chol in an eye lens lipid membrane can significantly reduce the hydrophobic environment near the membrane surface, and this reduction in hydrophobicity impairs and directly correlates with the previously discussed decrease in β_L_-crystallin binding to the membrane.

These results are similar to those that we previously found for the case of α-crystallin. In our prior studies, α-crystallin was also associated with eye lens-lipid membranes via hydrophobic interactions, and these interactions were further modulated by the lipid composition of each model lens-lipid membrane [24]. Moreover, adding Chol into the membrane showed similar effects, lowering the hydrophobicity and reducing the binding of α-crystallin with the membrane [24]. However, comparing the α-crystallin and β_L_-crystallin binding to the membranes, in the case of α-crystallin, the opposite lipid preference was found (i.e., having the highest MMSO in the SM-dominant MHLL membrane and the lowest binding in the PL-predominant MPLL and MMLL membranes). Although the general preference for lipids varies, α-crystallin binding also had reduced binding with an increase in the POPE content, similar to that reported in this study for β_L_-crystallin. Therefore, while the primary lipid that each crystallin binds to varies, it appears that both α- and β_L_-crystallins can bind to the eye lens-lipid membrane through hydrophobic interactions, and the addition of Chol into the membrane reduces the hydrophobicity near the membrane headgroup region ultimately diminishing the association of α- and β_L_-crystallin with the membrane. The results reported in this manuscript are significant, as they characterize the nature of the interactions of β_L_-crystallin with membranes and the role of Chol and lipid composition in β_L_-crystallin membrane binding, as well as modulation on the physical properties (mobility, order, and hydrophobicity) of membranes with β_L_-crystallin binding.

## 4. Materials and Methods

### 4.1. Materials

Fresh ~2-year-old bovine lenses were acquired from Pel-Freez Biologicals (Rogers, AZ, USA) and stored at −80° C until use. Cholesterol (Chol), sphingomyelin (SM), and phospholipids (PLs): 1-palmitoyl-2-oleoyl-sn-glycero-3-phosphatidylCholine (POPC), 1-palmditoyl-2-oleoyl-sn-glycero-3-phosphoethanolamine (POPE), and 1-palmitoyl-2-oleoyl-sn-glycero-3-phosphatidylserine (POPS) were obtained in chloroform from Avanti Polar Lipids, Inc. (Alabaster, AL, USA). Cholesterol analog cholestane spin-label (CSL), HEPES, Tris-HCl, NaN_3_, and sodium chloride (NaCl) were obtained from Sigma Aldrich (St. Louis, MO, USA). CSL spin-label was dissolved in chloroform, and the native β_L_-crystallin was extracted and purified from the bovine lens cortex and stored in HEPES buffer (10 mM HEPES, 100 mM NaCl, pH = 7.4), as described in Section 4.2. All preparations of β_L_-crystallin and the model lens lipid membranes, as well as associated studies into the β_L_-crystallin membrane interactions, were performed in HEPES buffer (10 mM HEPES, 100 mM NaCl, pH = 7.4).

### 4.2. Extraction and Purification of Native Bovine β_L_-Crystallin

A single ~2-year-old bovine lens was decapsulated, and the cortical and nuclear regions were further separated based on tissue consistency, as described previously [26]. Soluble proteins from the cortex of ~2-year-old bovine lens were separated using the protocol [4] described in our previous studies [26]. β_L_-Crystallin (low molecular weight β-crystallin) fraction was purified further using a methodology previously described [67]. Briefly, the cortical tissue was first homogenized in elution buffer (20 mM Tris-HCl, 150 mM NaCl, and 1 mM NaN_3_ with pH 7.9) to begin extracting soluble cortex proteins. Homogenized tissues were spun in a centrifuge (Beckman Coulter, Brea, CA, USA) at 18,000× *g* for 15 min at 4° C to separate the soluble cellular proteins from cellular debris. The supernatant containing proteins was removed and filtered using a 0.22 µm pore syringe filter. A total of 5 mL of filtered supernatant was loaded into an AKTA go protein purification system with a Hiload 16/600 Superose 6 pg gel filtration column for purification with size exclusion chromatography [26]. The solution was eluted at a 1 mL/min flow rate and monitored at a 280 nm absorbance to separate crystallin fractions. The fourth peak corresponded to β_L_-crystallin fractions [4], which were further purified using the Sephacryl S-200 HR column, as described previously [67]. Collected β_L_-crystallin solutions were then concentrated by centrifuging the solutions in Amicon Ultra-15 filters at 5000 RPM and 4 °C. The concentrated solutions were then dialyzed in a buffer (10 mM HEPES, 100 mM NaCl, pH 7.4) and stored at −80° C until further use. The purity of the β_L_-crystallin was confirmed using sodium dodecyl sulfate–polyacrylamide gel electrophoresis (SDS-PAGE). Furthermore, the concentration of the isolated β_L_-crystallin was determined by measuring the UV absorbance at 280 nm in triplicate. For the concentration calculations, an average molecular weight of 24.31 kDa and average extinction coefficient of 55,409 M^−1^ cm^−1^ was used, which were estimated from seven β_L_-crystallin subunits (βA1, βA2, βA3, βA4, βB1, βB2, and βB3) using the ProtParam tool on the Expasy server [68]. Previous studies show that β_L_-crystallin isolated from the bovine lens cortex is composed of these seven β-crystallin subunits [55]. The purified β_L_-crystallin was further used for the EPR measurements.

### 4.3. Preparation of Model Human, Porcine, and Mouse Lens-Lipid Membranes

The distinct compositions of the phospholipids and sphingolipids used to make the MHLL, MPLL, and MMLL membranes were acquired from a previous study [24]. For the Chol/MHLL membrane at a mixing ratio of 0 (without Chol), we used 66% SM, 11% POPC, 8% POPS, and 15% POPE. Moreover, for the Chol/MPLL membrane at a mixing ratio of 0, we used 29% SM, 35% POPC, 21% POPS, and 12% POPE, while 15% SM, 46% POPC, 17% POPS, and 17% POPE were used for the Chol/MMLL membrane at a mixing ratio of 0. To prepare the samples for each model lens-lipid membrane, the PLs and SM were mixed with a chloroform solution of CSL spin-label that was maintained at a concentration of 1 mol% in a mixture of Chol and lipids (SM, POPC, POPS, and POPE). The mixing ratios of the Chol/MHLL, Chol/MPLL, and Chol/MMLL membranes were maintained at 0 (without Chol) and 0.3 (with 23 mol% Chol). Following mixing, N_2_-gas was used to dry the mixtures to a volume of ~75 μL. Once dried, approximately 360 µL of warm (~50 °C) HEPES buffer (10 mM HEPES, 100 mM NaCl, pH = 7.4) was added to the remaining mixture. The rapid solvent exchange (RSE) method, described in our previous studies, was then used to prepare large multilamellar vesicles (LMVs) [69,70]. Probe tip sonication (Fisher Scientific, Hampton, NH, USA, Model 550) of the LMVs was then used for 20 cycles of 10 s sonication followed by 15 s of cooling on ice to prepare small unilamellar vesicles (SUVs). The detailed method for preparing SUVs using RSE and probe tip sonication has been described in our prior studies [25,28]. Each sample’s final concentration of Chol plus lipids (SM, POPC, POPS, and POPE) was maintained at 40 mM. The hydrodynamic radius of the SUVs for each sample was determined using dynamic light scattering (DLS) and further used in Section 4.4 for the calculations of the MSO by β_L_-crystallin.

Following the preparation of the SUVs, β_L_-crystallin was mixed at varying concentrations with fixed concentrations (11.4 mM) of Chol plus lipids (SM, POPC, POPS, and POPE) in a total volume of 70.0 μL for each of the Chol/MHLL, Chol/MPLL, and Chol/MMLL membranes. The β_L_-crystallin concentrations ranged from 0–52.6 μM, for the Chol/MHLL membrane, to 0–114.2 μM, for the Chol/MPLL and Chol/MMLL membranes. Our prior studies on α-crystallin membrane binding [24] showed that the binding saturations of α-crystallin to the model lens-lipid membranes was obtained with an α-crystallin concentration no greater than 52.6 μM, which is why this value was initially used for the β_L_-crystallin membrane binding studies. However, while saturation was achieved in the MHLL membrane, there was no binding saturation of β_L_-crystallin to the MPLL or MMLL membranes at a concentration of 52.6 μM. Multiple experiments were conducted at increasing β_L_-crystallin concentrations for both animal models until the β_L_-crystallin association appeared saturated at 114.2 μM, so the MPLL and MMLL membranes were incubated with higher concentrations of β_L_-crystallin than those used in the MHLL membrane. The membranes were prepared without Chol or with a Chol/lipid mixing ratio of 0.3. The membranes with a fixed lipid plus Chol concentration that were mixed with varying concentrations of β_L_-crystallin were then incubated at 37 °C with gentle shaking (150 rpm) for 16 h in a benchtop incubator (Corning Inc., Corning, NY, USA) to allow for the saturation binding of β_L_-crystallin with each model lens-lipid membrane, similarly as previously described in our prior studies on α-crystallin membrane binding [25,27,28]. The experiments were repeated at least three times, and at least three different preparations of the samples were used for repetitive experiments.

### 4.4. Electron Paramagnetic Resonance (EPR) Spin-Labeling Method for Investigation of β_L_-Crystallin Binding to Models of Human and Animal Eye Lens-Lipid Membranes

The incubated samples were loaded into a gas-permeable methylpentene polymer (TPX) capillary for continuous wave (CW) electron paramagnetic resonance (EPR) using an X-band Brucker ELEXSYS 500 spectrometer. Measurements were taken at either 37 °C to obtain information on β_L_-crystallin binding with the membranes and the consequential effects on the membrane’s mobility and order near the headgroup regions with β_L_-crystallin binding or at −165 °C to obtain the hydrophobicity near the membrane head group region with β_L_-crystallin binding. All measurements taken at 37 °C used a 0.8 mm internal diameter (i.d.) TPX capillary, while for the measurements at −165 °C, a 1.0 mm i.d. TPX capillary is used to improve the signal-to-noise ratio. At both temperatures, a constant stream of N_2_-gas was used to deoxygenate the samples and maintain the temperature; however, the experiments at −165 °C also required liquid N_2_ to maintain the low temperature. The EPR spectra from the measurements at 37 °C were taken with a modulation amplitude of 1.0 G and an incident microwave power of 8.0 mW.

Moreover, at −165 °C, the EPR spectra were taken with a modulation amplitude of 2.0 G and incident microwave power of 2.0 mW. As described in our previous papers [22,25,27,28], these EPR spectra are produced from the CSL spin-label in the membrane, where CSL has a structure similar to the Chol molecule, with the hydroxyl group of Chol replaced with a free radical containing a nitroxide moiety, and the CSL spin-label integrates into the membrane near the headgroup region (see Figure 6 in Ref. [23] for the structure of Chol and CSL and their location on the lipid bilayer). The EPR spectra of the CSL spin-label in the membrane with and without β_L_-crystallin binding were normalized to the peak-to-peak intensity of each spectrum’s central line to obtain information regarding the interactions of β_L_-crystallin with the membrane.

**Figure 6:**
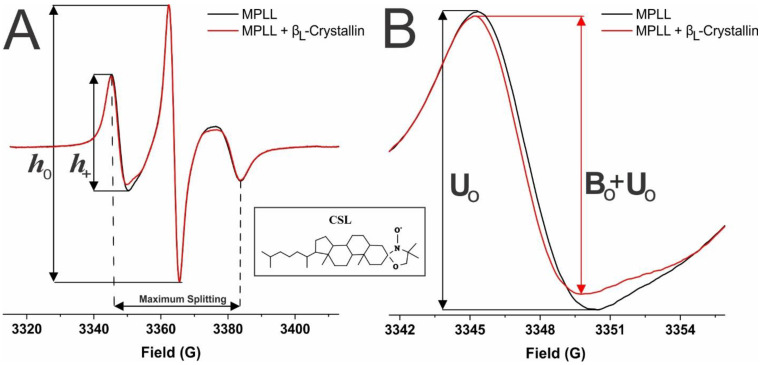
(**A**) EPR spectra of CSL in MPLL membranes taken at 37 °C in the absence of β_L_-crystallin (black) and with 114.2 μM β_L_-crystallin (red). The ratio of the peak-to-peak intensity of the low-field line (h_+_) and the central line (h_0_) provides the mobility parameter (h_+_/h_0_). The horizontal distance between the low- and high-field lines provides the maximum splitting. (**B**) Magnified image of the low-field line of the EPR spectra shown in (**A**), representing the unbound (U_0_) and unbound plus bound (U_0_ + B_0_) contributions. The change in the peak-to-peak intensity of the low-field line of the EPR spectra was used to calculate the percentage of the membrane surface occupied (MSO) by β_L_-crystallin and the binding affinity (K_a_). Shown boxed between (**A**,**B**) is the chemical structure of the CSL spin-label. The nitroxide moiety, displayed on the right of the structure, integrates into the membrane near the surface (below the headgroup region), which allows us to probe the interactions of β_L_-crystallin with the membrane.

Shown in Figure 6A is a demonstrative EPR spectra, taken at 37 °C for the CSL spin-label in the membranes not containing β_L_-crystallin (black) and containing 114.2 μM β_L_-crystallin (red). From these spectra, we can obtain information on the percentage of membrane surface occupied (MSO), binding affinity (K_a_), mobility parameter, and maximum splitting. As shown by the dotted lines, the distance in peaks from the low-field to the high-field lines gives us the values for the maximum splitting, which provides the amplitude of the wobbling motion of the long axes of the spin-labels in the membranes and is related to the order parameter [25,28,57,58,59]. Moreover, as seen by the solid lines, the ratio of the peak-to-peak height of the low-field line (h_+_) and the central line (h_0_) provides us with the value for the mobility parameter, which supplies information regarding the orientational and rotational dynamics of the spin-labels in membranes [25,28]. Shown in Figure 6B are zoomed in low-field EPR lines of the spectra displayed in Figure 6A, and the red line in Figure 6B shows that β_L_-crystallin binding to the MPLL membrane decreased the peak-to-peak intensity of the low-field EPR line relative to the control membrane (MPLL membrane without β_L_-crystallin, shown by the black line). Previously, we have observed a similar decrease in the peak-to-peak intensity of a low-field EPR line when α-crystallin binds with model lens-lipid membranes [24].

As further depicted in Figure 6B, the low-field line of the EPR spectra from the control membranes without β_L_-crystallin was used as an unbound contribution (U_0_), and the low-field line of the EPR spectra with β_L_-crystallin binding was used as an unbound plus bound (U_0_ + B_0_) contribution. This information was then used to calculate the percentage of CSL spin-labels near the surface of the membrane affected by β_L_-crystallin binding using a method described previously [25,27,28]:

(1) $$\%\;\mathrm{CSL}\;\text{spin-labels}\;\mathrm{affected}=\left\{\frac{U_{0}-(U_{0}+B_{0})}{U_{0}}\right\}\times 100\%$$

DLS measurements taken on a DynaPro instrument (Wyatt Technology Corp., Santa Barbara, CA, USA) using regularization methods (Dynamics software, version 7) were used to determine the hydrodynamic radius of the SUVs for all individual MHLL, MPLL, and MMLL membrane samples. Individual radius data were used to calculate the MSO by β_L_-crystallin for each sample. The DLS measurements indicated that the radius of the MHLL vesicles was ~64 nm and the MPLL and MMLL vesicles was ~41 nm. Based on these radii, ~53% of the CSL molecules were on the outer surface of the MHLL membrane, and ~55% of the CSL molecules were on the outer surface of the MPLL and MMLL membranes. As the only CSL spin-labels affected by the binding of β_L_-crystallin were those on the outer surface, the corrected percentage of CSL spin-labels affected by β_L_-crystallin or MSO by β_L_-crystallin was estimated by multiplying Equation (1) by the corrections factor. A correction factor of 100/53 was used for the MHLL samples, and a correction factor of 100/55 was used for the MPLL and MMLL samples to estimate the corrected % CSL spin-label affected or the MSO by β_L_-crystallin, as performed earlier in our previous studies [24,25,27,28]. An example calculation for the MPLL membrane with the correction factor reads as follows:

(2) $$\%\;\mathrm{Membrane}\;\mathrm{surface}\;\mathrm{occupied}\;(\mathrm{MSO})=(\%\;\mathrm{CSL}\;\text{spin-labels}\;\mathrm{affected})\;\times \;\left(\frac{100}{55}\right)$$

This MSO by β_L_-crystallin for each eye lens-lipid model membrane was used to calculate the K_a_ of β_L_-crystallin to the membrane based on the procedures described in our previous studies [24,25,27,28]. In the EPR method, the estimation of the MSO is based on the relative decrease in the peak-to-peak intensity of the low-field line due to the association of β_L_-crystallin with the membrane. The calculated MSO value provides information regarding the percentage of outer membrane surface occupied by β_L_-crystallin and gives us a quantitative method for analyzing protein binding. The determined MSO by β_L_-crystallin for each sample was further plotted as a function of the β_L_-crystallin concentration, and the data were fitted using a one-site ligand binding model in GraphPad Prism (San Diego, CA) to calculate the binding affinity (K_a_), as explained in our previous studies [25,27,28].

**Figure 7:**
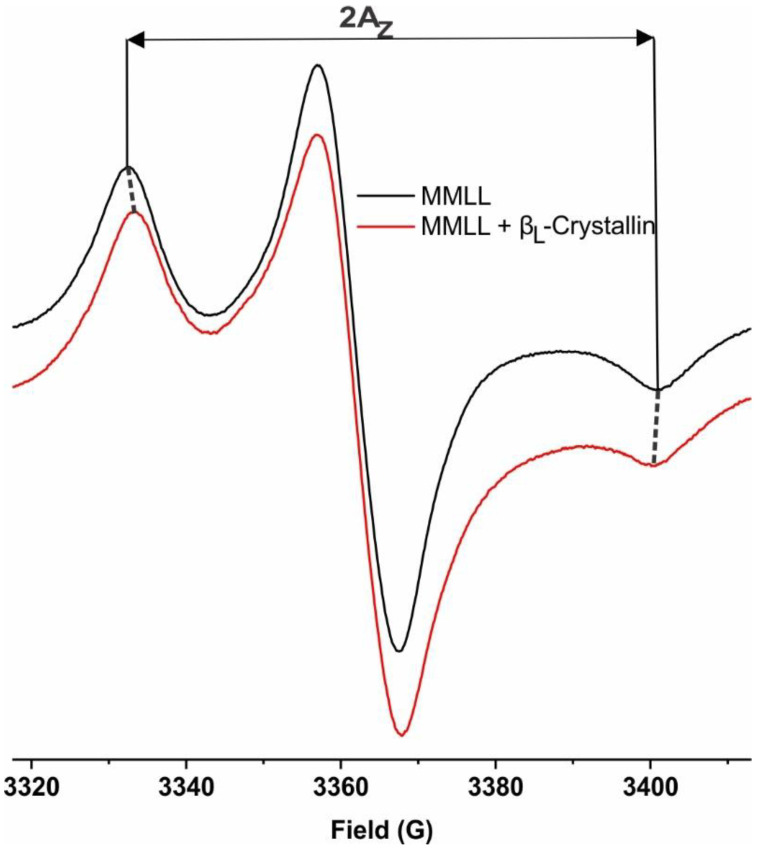
EPR spectra of CSL in the MMLL membranes taken at approximately −165 °C in the absence of β_L_-crystallin (black) and with 114.2 μM β_L_-crystallin (red), showing the horizontal distance between the low-field and high-field lines, being used to measure 2A_Z_, which is a measure for hydrophobicity.

The z-component of the hyperfine interaction tensor (A_z_) for the CSL spin-labels in the MHLL, MPLL, and MMLL membranes was measured from the EPR spectra recorded for the samples frozen at approximately −165 °C. Liquid nitrogen was used to maintain the temperature at approximately −165 °C. Figure 7 shows the horizontal distance between the low-field line and the high-field line of the EPR spectra of the MMLL membranes without and with β_L_-crystallin taken at approximately −165 °C, resulting in 2A_z._ The 2A_z_ value is a measure of the hydrophobicity [24,47,57,58,60,71]. The higher the 2A_z_ value from the CSL spin-label in a membrane, the lower the hydrophobicity near the headgroup region of the membrane [22,23,24,57].

### 4.5. Statistics

All results are presented as the mean ± standard deviation (σ) with at least three independent experiments. We evaluated the statistically significant differences in the MMSO, K_a_, mobility parameter, maximum splitting, and hydrophobicity values using the Student’s *t*-test. A value of *p* ≤ 0.05 was considered statistically significant.

## Data Availability

The data presented in this study are available in the article.

## Abbreviations

EPR	Electron paramagnetic resonance
MHLL	Model of human lens-lipid
MPLL	Model of porcine lens-lipid
MMLL	Model of mouse lens-lipid
MSO	Percentage of membrane surface occupied
MMSO	Maximum percentage of membrane surface occupied
CSL	Cholesterol analog cholestane spin-label
PL	Phospholipid
SM	Sphingomyelin
Chol	Cholesterol
